# Research on the Clinical Practical Use of Pivoxil-Conjugated Antibodies and the Risk of Carnitine Deficiency Using Real-World Data

**DOI:** 10.3390/children11020150

**Published:** 2024-01-24

**Authors:** Kaho Suzuki-Yoshida, Kosuke Nakano, Masayoshi Nakakuni, Naoko Deguchi, Seiji Mitsui, Shinji Kobayashi, Akimasa Yamatani, Miki Akabane

**Affiliations:** 1 Graduate School of Pharmaceutical Sciences, Meiji Pharmaceutical University, Tokyo 204-0004, Japan; 2 Department of Pharmacy, National Center for Child Health and Development, Tokyo 157-8535, Japan; 3 MSD K.K., Tokyo 102-8667, Japan; 4 Clinical Research Center, National Center for Child Health and Development, Tokyo 157-8535, Japan; 5 Nature Insight Co., Ltd., Tokyo 101-0021, Japan

**Keywords:** pivoxil-conjugated antibodies, real-world data, children, carnitine deficiency

## Abstract

In Japan, pivoxil-conjugated antibodies (PVs) are commonly used to treat infections. However, carnitine deficiency is a known adverse drug reaction associated with PV treatment. This study aimed to research the practical use of PV and assess the risk of carnitine deficiency in patients receiving PV compared to their amoxicillin (AM)-treated counterparts. The Pediatric Medical Information Collection System (P-MICS) served as the data source for this study. The study cohort comprised patients aged 0–15 years prescribed PV between April 2016 and March 2021. Data on the actual PV prescriptions were extracted for each patient. To evaluate the risk of carnitine deficiency, adverse events (AEs) were defined as carnitine deficiency and its associated symptoms. Propensity score matching was employed to compare the AE incidence between the PV and AM groups. The number of cases of PV prescriptions decreased year-on-year between 2016 and 2021, and >80% of prescriptions were dispensed in the clinic. The propensity score matching analysis demonstrated no statistically significant difference in the incidence of carnitine deficiency and its associated symptoms between the PV and AM groups. Our findings suggest that the risk of carnitine deficiency in children treated with PV is not significantly higher than that associated with other antibiotics.

## 1. Introduction

In Japan, pivoxil-conjugated antibodies (PVs), which are combined with pivalic acid for enhanced bioavailability, are widely employed for treating infections like otitis media [1]. Carnitine deficiency is known as a potential adverse drug reaction associated with PV usage. This risk stems from the conjugation of carnitine with pivalic acid during excretion, leading to the depletion of carnitine stores. This effect is particularly pronounced in children, who naturally have lower blood carnitine levels. Severe cases of carnitine deficiency associated with PV have been reported, manifesting as hypoglycemia, convulsion, and other serious adverse drug reactions [2,3]. Although carnitine deficiency caused by PV usage is a secondary carnitine deficiency, it is known that primary carnitine deficiency caused by congenital genetic abnormalities also exhibits similar symptoms to secondary carnitine deficiency [4]. In April 2012, the Pharmaceuticals and Medical Devices Agency (PMDA) issued a “Request for Proper Use of Drugs”, highlighting the potential risks of PV use in children [1]. This was followed by a similar cautionary statement released by the Japanese Society of Pediatrics in July 2019 [5].

While case reports have documented the actual prescription of PV to children, the occurrence of carnitine deficiency related to its administration, and subsequent treatment strategies [6,7,8,9,10], a large-scale survey has not yet been conducted. Additionally, although a report explored the link between PV and hypoglycemia in children [11], no large-scale studies have directly evaluated the risk of carnitine deficiency itself compared to other antibiotics. Consequently, a research study is essential to clarify the potential risk of carnitine deficiency associated with PV administration in children.

Therefore, (1) a PV prescription survey, (2) an exploration of the relationship between the onset of carnitine deficiency due to PV and the number of days of administration and test/treatment at the time of onset, and (3) an investigation of the risk of carnitine deficiency in patients treated with PV were conducted by using the Pediatric Medical Information Collection System (P-MICS), built by the National Center for Child Health and Development [12,13].

## 2. Materials and Methods

### 2.1. Database

This research utilized the P-MICS database. P-MICS was established to elucidate actual drug usage in pediatrics and contribute to promoting the safe and appropriate use of medications within this population as part of the Pediatric Drugs Information Collection Network Development Project subsidized by the Ministry of Health, Labor, and Welfare.

The pediatric DB was established for the purposes of clarifying the actual use status of drugs in the pediatric field and contributing to the proper use and safety measures of pediatric drugs in the project for the improvement of the pediatric and drug information collection network. Comprehensive data were collected from electronic medical records across the nation (as of July 2023). This included data from 11 pediatric hospitals and 30 clinics primarily specializing in pediatric care. P-MICS houses a unique collection of medical information for approximately 710,000 children in Japan. This non-modifiable database includes data points such as disease names, prescription/injection data, and laboratory results, facilitating data analysis. Notably, P-MICS is the only electronic medical record database in Japan dedicated to pediatric healthcare, collecting information from both hospitals and clinics across the nation. Given that antibacterial drugs are frequently prescribed in both hospitals and clinics, the P-MICS database offers a valuable representation of the actual prescription landscape of these medications. Therefore, P-MICS was deemed a suitable data source for the present study.

### 2.2. Study Design

For the PV prescription survey, all patients within the P-MICS database between 1 April 2016 and 31 March 2021, were extracted for analysis. This excluded patients with missing information regarding sex and/or birth date. From the analysis dataset, patients aged <15 years who received a prescription for PV, including cefditoren pivoxil, cefcapene pivoxil hydrochloride, cefteram pivoxil, and tebipenem pivoxil, were identified using their ATC codes (J01DD16, J01DD17, J01DD18, J01DH06) and extracted as the study population. Furthermore, we investigated the potential association between carnitine deficiency onset following PV administration, the duration of treatment, and diagnostic/therapeutic procedures implemented at the time of onset.

To assess the risk of carnitine deficiency in patients treated with PV, we conducted a retrospective cohort observational study using real-world data from the P-MICS database. A retrospective study design was employed to identify a cohort of patients aged <15 years from the P-MICS database. Patients who received prescriptions for either PV (ATC code: J01CA04) or amoxicillin (AM), the comparator antibiotic, for a 5-year period from 1 April 2016 to 31 March 2021, were included. Propensity score matching was employed to create two balanced groups: an observed group consisting of patients prescribed PV as observed cases and a comparator group consisting of patients prescribed AM. This facilitated a comparative analysis of the number and incidence of adverse events (AEs) between the two groups. AM was selected as the comparator antibiotic due to its well-established first-line status for treating infections such as otitis media, pharyngitis, and upper respiratory tract inflammation, which are also indications for PV, as recommended by the Manual of Antimicrobial Stewardship [14].

### 2.3. Definition of Adverse Events (AEs)

Disease names recorded within a 90-day window following the last administration of PV or AM were extracted. Cases involving carnitine deficiency and its associated symptoms of hypoglycemia, altered consciousness [5], and convulsions were identified and classified as “disease name data related to carnitine deficiency”. These specific diagnoses were coded according to the ICD10 system (E713, R402, R55, G253, G400, G839, E15, E160, E161, E162, P703, P704) and subsequently used for analysis.

### 2.4. Exclusion Criteria

To examine the relationship between the onset of carnitine deficiency due to PV and the number of days of administration and test/treatment at the time of onset, subjects were excluded if they did not have information from 180 days before the first dose of PV and 90 days after the last dose of PV. Additionally, subjects with a similar AE prior to the first PV dose or those who received levocarnitine treatment before the initial PV dose were excluded from the analysis.

To refine the investigation of carnitine deficiency risk in PV-treated patients, an additional exclusion criterion was implemented. Subjects who received the co-administration of both PV and AM were excluded from the analysis to isolate the potential effects of PV on carnitine deficiency.

### 2.5. Analysis Methods

To assess the risk of carnitine deficiency associated with PV use, the dataset was divided into two distinct groups: PV and AM groups. These groups formed the basis for generating the initial datasets (original datasets) for subsequent analysis. To ensure balanced comparisons between the PV and AM groups, a matched dataset was created using propensity score matching. Logistic regression was employed for the one-to-one non-restoration matching of propensity scores, resulting in an analysis dataset with an equal number of subjects in both groups. For the propensity score calculation model, the drug group (PV or AM) was designated as the dependent variable. Independent variables included site category, sex, age, age category, number of diseases diagnosed during the 180 days preceding administration initiation, number of hospital visits during the same period (180 days), number of prescribed medications during 180 days before initial administration, presence/absence of preexisting diseases (specific pediatric chronic diseases and diseases of potentially impacting carnitine deficiency occurrence; see Table S1), and presence/absence of prescription for medications potentially impacting carnitine deficiency occurrence (Table S2) before the prescription start date.

The primary outcome was defined as the occurrence of AEs based on disease name data recorded within 90 days after the last administration of the medication. The number and incidence of AEs were then compared between the PV and AM groups. Data extraction, processing, and statistical analysis were conducted using SAS 9.4 TS Level 1M7 software (Version: 9.04.01M7P080520).

## 3. Results

### 3.1. Pivoxil-Conjugated Antibody (PV) Prescription Survey

**Figure 1:**
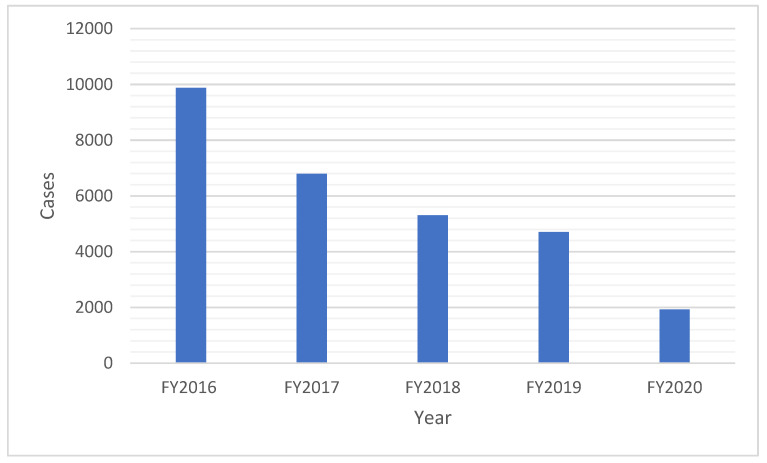
Number of antibiotic prescriptions containing a pivoxil-conjugated antibody (PV) by year.

We examined the yearly trends in PV prescriptions during the study period (April to March of the following year; see Figure 1). Notably, a consistent year-on-year reduction in the number of prescriptions was observed since the survey’s initiation in 2016.

Further investigation focused on analyzing the age and site categories associated with PV prescriptions. This analysis revealed that toddlers (1–7 years) and children (7–15 years) accounted for over 90% of all PV prescriptions, regardless of the setting (clinics or hospitals; see Table 1). Additionally, clinics dispensed roughly 80% of the total number of prescriptions.

Furthermore, we compared the prescription volume for each of the four commercially available PV drugs in Japan. Cefcapene pivoxil hydrochloride hydrate and cefditoren pivoxil jointly accounted for approximately 90% of all prescriptions (Table 2). The median prescription duration remained consistent within a range of 4–5 days, suggesting no significant variations in administration length. This finding indicates that the drug was typically prescribed for a short period.

### 3.2. Relationship between Pivoxil-Conjugated Antibody (PV)-Induced Carnitine Deficiency Onset, Administration Days, and Onset Test/Treatment

Initially, we investigated the relationship between the onset of carnitine deficiency associated with PV and the duration of drug administration. While the number of subjects with AEs during the treatment period did not exhibit significant differences, we observed variations in the timing of AE onset, with some subjects experiencing AEs as early as Day 6 and others as late as Day 762 (Table 3). Furthermore, we analyzed the cumulative number of treatment days from the first PV dose to the onset of AEs. Notably, most subjects experienced AEs within a cumulative treatment period of 4 weeks or less (Table 4).

Further analysis focused on exploring the diagnostic and therapeutic interventions implemented at the time of AE onset among the subjects with AEs potentially related to carnitine deficiency. The investigation revealed a limited number of cases confirmed as carnitine deficiency, with many diagnoses instead indicating hypoglycemia. Notably, no subjects experienced consciousness disturbances or syncope episodes (Table 5).

By analyzing the specific tests and treatments administered at the time of AE onset, we found that carnitine testing was performed in a subset of confirmed carnitine deficiency cases, while carnitine replacement therapy was implemented in more than 70% of these patients. Notably, neither carnitine testing nor carnitine replacement therapy was implemented in any of the hypoglycemia-diagnosed patients.

### 3.3. Investigation of the Risk of Carnitine Deficiency in Pivoxil-Conjugated Antibody (PV)-Treated Patients

Data extraction yielded a total of 47,004 subjects for inclusion in the study. After matching, 11,107 subjects were included in each arm, resulting in balanced groups of 19,110 subjects in the PV arm and 27,894 subjects in the AM arm.

Prior to matching, the baseline characteristics between the PV and AM groups exhibited significant variations depending on the site category. Notably, the proportion of subjects with hospital visits differed considerably, with 18.7% in the PV group and 73.4% in the AM group (standardized difference: ±1.31; see Table 6). Aside from site category, no other baseline characteristics exhibited significant differences between the PV and AM groups (standardized difference: ≥±0.4, details provided in Table S3). The matching procedure successfully eliminated all significant discrepancies in the baseline characteristics between the PV and AM groups.

Table 7 presents the results of propensity score matching, with AM serving as the control group. Hypoglycemia emerged as the most prevalent AE, followed by carnitine deficiency. Notably, the disturbance of consciousness was exclusively observed in the AM group, while no cases of convulsions were reported in either group.

## 4. Discussion

Our analysis of PV prescription data collected through a continuous survey starting in 2016 revealed a significant and sustained decrease in the number of prescriptions over time (Figure 1). This decline may be partially attributed to the dissemination of the principles on how to use antibacterial drugs in the Antimicrobial Resistance Action Plan in 2016 [15] and the promotion of the proper use of antibacterial drugs by specific premiums incentivizing proper antibiotic prescribing practices, both for general and pediatric populations, within the medical service fee structure starting in 2018 [16,17]. Furthermore, a significant decline in prescriptions (50%) was observed in fiscal 2020 compared to 2019. This decrease is likely attributed to reduced healthcare visits due to COVID-19 and behavioral changes leading to fewer non-COVID-19 infections [18,19].

We found that PV prescriptions were primarily issued in clinics, predominantly for children aged 3–15 years (Table 1). Notably, cefcapene pivoxil hydrochloride hydrate and cefditoren pivoxil accounted for approximately 90% of all PV prescriptions among the four available drugs in Japan (Table 2). All PV formulations launched in Japan include pediatric-friendly granules, with no variation in dosage form [20,21,22,23]. This suggests that the substantial prescription volume of these two drugs is likely attributed to their earlier market entry and a wider range of approved indications compared to the other two medications.

Additionally, our survey revealed that carnitine deficiency-related AEs occurred not only during the initial phase of treatment but also persisted throughout long-term therapy (Table 3 and Table 4). This finding is consistent with the observations reported in previous studies [6,7,8,9,10,24]. Therefore, a careful consideration of the risk given the potential for AEs to manifest throughout the entire treatment course is essential when administering PV.

Conversely, among the PV-treated subjects diagnosed with carnitine deficiency, carnitine testing was conducted in some cases, and carnitine replacement therapy was administered to over 70% of patients. Notably, neither carnitine testing nor replacement therapy was implemented for any hypoglycemia-diagnosed subjects. Given these findings, the potential association between carnitine deficiency and PV-associated hypoglycemia requires further attention (Table 5). Also, it is known that pivalic acid, a metabolite of PV, can also cause an increase in isovalerylcarnitine [25], which may lead to false positives in identifying isovaleric acidemia. Therefore, careful consideration should be given when using the results of newborn screening and tests carried out during PV administration to differential diagnosis of isovaleric acidemia.

Our investigation into the potential for carnitine deficiency in PV-treated patients revealed no statistically significant differences in carnitine deficiency occurrence or associated symptoms compared with the control group treated with AM (Table 7). These findings suggest that the current risk of carnitine deficiency in children receiving PV treatment is not considerably higher than that of other antibiotics. This observation is likely attributed to the heightened awareness of potential carnitine deficiency in PV-treated patients, driven by cautionary messages from the PMDA and the Japanese Pediatrics Society, coupled with efforts promoting appropriate PV use [1,5]. A significantly higher incidence of hypoglycemic events was observed in patients receiving PV treatment than in patients receiving other oral β-lactam antibiotics based on health insurance claims analyzed in a previous study investigating PV-associated hypoglycemia [11]. The incidence of hypoglycemia-related AEs in our study was lower than that observed in that previous report [11]. This discrepancy may be attributed to three key factors. First, the previous study utilized data predating the call for attention to proper PV usage, while our research solely employs data collected after this intervention. Second, our study defines AEs as the occurrence of carnitine deficiency and associated symptoms, including hypoglycemia, disturbed consciousness, and convulsions. In contrast, the previous study included subjects solely based on the diagnosis of hypoglycemia or the receipt of glucose therapy. Third, our analysis excludes subjects who received carnitine replacement therapy during the observation period. This exclusion ensures that the results reflect the genuine occurrence of AEs arising from PV use in the context of current medical practice, given that the large-scale investigation focused on identifying individuals with unmanaged carnitine deficiency symptoms.

Our study has several limitations. The sample size employed in the propensity score matching analysis (11,107 subjects per group after matching) might have been inadequate to identify the incidence of AEs. As such, conducting a similar investigation with a larger sample size is crucial to corroborate the current findings. Significant differences were observed between the PV and AM groups regarding the site category within baseline patient characteristics, with a higher proportion of hospital prescriptions in the AM group (Table 6). In general, the severity of disease encountered in hospital settings tends to be higher than in clinics. Consequently, treatments administered for these more severe conditions may inherently carry a greater risk of AEs than treatments for less severe diseases. Despite incorporating covariates that may influence patient severity, such as site category and the number of pretreatment diseases, residual differences in treated disease severity may have persisted between groups, even after matching. Additionally, a wider range of indications exist for AM than for PV [20,21,22,23,26]. This disparity in potential uses may have contributed to the observed difference in severity between the two groups. Additionally, the database used in our study, P-MICS, does not include the results of the newborn screening panel in which tests for genetic carnitine deficiency are included. Therefore, the possibility cannot be denied that patients with genetic carnitine deficiency were included in the analysis. However, patients with genetic carnitine deficiency usually receive chronic levocarnitine treatment, and, in this study, patients who received levocarnitine treatment before the initial PV/AM were excluded from the analysis. Therefore, it is considered that it is unlikely that patients with genetic carnitine deficiency were included in this study, and this is unlikely to affect the interpretation of the results.

Utilizing a children-specific database provided access to significantly more detailed patient background information than traditional health insurance claim databases. These comprehensive data, including the number of pretreatment diseases, number of hospital visits, number of prescribed medications, and presence/absence of potential comorbidities/medications impacting carnitine deficiency, facilitated the inclusion of a larger number of covariates in the analysis. Therefore, propensity score matching enabled us to obtain results with a reduced influence of confounding factors not accounted for by the included covariates, thereby approximating the outcomes observed in actual clinical settings. Nonetheless, as mentioned above, the possibility of an insufficient sample size for the reliable detection of AE incidence remains, and, therefore, further investigation with accumulated data is necessary to confirm these findings.

## 5. Conclusions

Based on the investigation into the potential for carnitine deficiency in PV-treated patients, the current risk of carnitine deficiency in children receiving PV treatment is not considerably higher than that of other antibiotics.

However, our study has several limitations. The sample size employed in the propensity score matching analysis might have been inadequate to identify the incidence of AEs. Also, despite incorporating covariates that may influence patient severity, residual differences in treated disease severity may have persisted between the PV and AM groups, even after matching.

Utilizing a children-specific database provided access to significantly more detailed patient background information, thereby approximating the outcomes observed in actual clinical settings. However, the possibility of an insufficient sample size for the reliable detection of AE incidence remains, and, therefore, further investigation with accumulated data is necessary to confirm these findings.

## Figures and Tables

**Table 1:** Number of PV prescriptions by dispensing site and age group.

Variables	Categories	All *n* (%)	Clinic *n* (%)	Hospital *n* (%)
Newborn	<4 weeks	8 (0.0)	5 (0.0)	3 (0.0)
Infant	≥4 weeks to <1 year	1626 (5.7)	358 (1.3)	1267 (4.4)
Toddler	≥1 year to <7 years	17,067 (59.6)	2841 (9.9)	14,216 (49.7)
Children	≥7 years to <15 years	9931 (34.7)	2379 (8.3)	7544 (26.4)
Total		28,613 (100.0)	5583 (19.5)	23,030 (80.5)

**Table 2:** Number and duration of each PV prescription.

Drug Name	Cases (*n* = 30,032) *n* (%)	Duration of Dosing (Days)		
		Mean (SD)	Minimum	Maximum
Cefditoren Pivoxil	17,557 (58.5)	7.01 (11.12)	1	753
Cefcapene Pivoxil Hydrochloride Hydrate	9228 (30.7)	6.43 (10.18)	1	429
Cefteram Pivoxil	2149 (7.2)	6.66 (6.67)	1	100
Tebipenem Pivoxil	1098 (3.7)	8.20 (6.90)	1	70

SD, standard deviation.

**Table 3:** Days to carnitine deficiency-related adverse event (AE) onset after first PV dose.

Duration	Cases (*n* = 22,364) *n* (%)	Days from First PV Dosing to AEs (Days)		
		Mean (SD)	Minimum	Maximum
0–6 days	8 (0.04)	2.3 (1.8)	1	6
1–4 week	15 (0.07)	19.5 (9.7)	7	34
5–12 week	16 (0.07)	60.5 (14.9)	36	78
13–24 week	13 (0.06)	133.3 (24.3)	96	166
≤25 weeks	6 (0.03)	305.8 (224.5)	181	762

SD, standard deviation; PV, pivoxil-conjugated antibody.

**Table 4:** Cumulative days from carnitine deficiency-related adverse event (AE) onset (PV first dose).

Duration	Cases (*n* = 22,364) *n* (%)	Days from First PV Dosing to AEs (Days)		
		Mean (SD)	Minimum	Maximum
0–6 days	30 (0.13)	4.0 (1.6)	1	6
1–4 week	26 (0.12)	12.5 (6.6)	7	32
5–12 week	0	NA	NA	NA
13–24 week	*	*	*	*
≤25 weeks	0	NA	NA	NA

* For one or two cases, the number of cases was masked to protect personal information, following Pediatric Medical Information Collection System (P-MICS) guidelines. SD, standard deviation; PV, pivoxil-conjugated antibody; NA, not applicable.

**Table 5:** Adverse event (AE) cases associated with carnitine deficiency and related tests/treatments at onset.

Category	Cases (*n* = 22,364) *n* (%)	Test (*n*)						Treatment (*n*)	
		Acylcarnitine Test		Free Carnitine Test		Carnitine Serum Test		Carnitine Supplementation	
		Yes	No	Yes	No	Yes	No	Yes	No
Carnitine deficiency	14 (0.06)	5	9	5	9	*	*	11	3
Hypoglycemia	44 (0.20)	0	44	0	44	0	44	0	44
Disturbance of consciousness	0	0	0	0	0	0	0	0	0
Convulsion	0	0	0	0	0	0	0	0	0

* For one or two cases, the number of cases was masked to protect personal information, following Pediatric Medical Information Collection System (P-MICS) guidelines.

**Table 6:** Main baseline patient characteristics.

Characteristics	Original Cohort (*n* = 47,004)				Matched Cohort (*n* = 22,214)			
	PV Group (*n* = 19,110)	AM Group (*n* = 27,894)	*p*-Value	Std Diff	PV Group (*n* = 11,107)	AM Group (*n* = 11,107)	*p*-Value	Std Diff
Site category								
Hospital (%)	3582 (18.74)	20,469 (73.38)	<0.0001	−1.310	3500 (31.51)	3713 (33.43)	0.0023	−0.046
Clinic (%)	15,528 (81.26)	7425 (26.62)	<0.0001	1.310	7607 (68.49)	7394 (66.57)	0.0023	0.046
Gender								
Male (%)	10,271 (53.75)	15,896 (56.99)	<0.0001	−0.065	6485 (58.39)	6134 (55.23)	<0.0001	0.064
Female (%)	8839 (46.25)	11,998 (43.01)	<0.0001	0.065	4622 (41.61)	4973 (44.77)	<0.0001	−0.064
Age								
Mean (SD)	5.61 (3.95)	4.56 (3.56)	<0.0001	0.279	6.21 (4.11)	5.15 (3.70)	<0.0001	0.279
Minimum	0	0	<0.0001	0.279	0	0	<0.0001	0.279
Maximum	14	14	<0.0001	0.279	14	14	<0.0001	0.279
Newborn (%)	5 (0.03)	41 (0.15)	<0.0001	−0.041	5 (0.05)	3 (0.03)	<0.0001	0.006
Infant (%)	1032 (5.40)	2365 (8.48)	<0.0001	−0.121	606 (5.46)	680 (6.12)	<0.0001	−0.026
Toddler (%)	10,782 (56.42)	17,976 (64.44)	<0.0001	−0.165	5594 (50.36)	6724 (60.54)	<0.0001	−0.209
Children (%)	7291 (38.15)	7512 (26.93)	<0.0001	0.241	4902 (44.13)	3700 (33.31)	<0.0001	0.233
Number of clinical site visits								
Mean (SD)	3.19 (7.44)	4.50 (10.45)	<0.0001	−0.144	3.83 (8.76)	4.05 (8.67)	0.0552	−0.025
Minimum	0	0	<0.0001	−0.144	0	0	0.0552	−0.025
Maximum	176	180	<0.0001	−0.144	171	170	0.0552	−0.025
Number of prescribed drugs								
Mean (SD)	4.57 (6.15)	5.07 (8.19)	<0.0001	−0.069	5.33 (6.73)	5.09 (7.56)	0.0134	0.033
Minimum	0	0	<0.0001	−0.069	0	0	0.0134	0.033
Maximum	108	116	<0.0001	−0.069	96	116	0.0134	0.033
Number of disease names								
Mean (SD)	2.52 (3.04)	3.62 (4.71)	<0.0001	−0.279	2.82 (3.30)	2.93 (3.22)	0.0096	−0.029
Minimum	0	0	<0.0001	−0.279	0	0	0.0096	−0.029
Maximum	49	65	<0.0001	−0.279	49	47	0.0096	−0.029

SD, standard deviation; PV, pivoxil-conjugated antibody; AM, amoxicillin; Std diff, standardized difference.

**Table 7:** Results of the propensity score matching analysis for adverse events (AEs).

Category		AEs Occurred (*n* = 11,107) n (%)	AEs not Occurred (*n* = 11,107) n (%)	*p*-Value	OR (95%IC)
Carnitine deficiency	PV group	6 (0.05)	11101 (99.95)	0.3171	0.60 (0.22–1.65)
	AM group	10 (0.09)	11097 (99.91)	0.3171	0.60 (0.22–1.65)
Hypoglycemia	PV group	21 (0.19)	11086 (99.81)	0.386	0.78 (0.44–1.38)
	AM group	27 (0.24)	11080 (99.76)	0.386	0.78 (0.44–1.38)
Disturbance of consciousness	PV group	0 (0.00)	11107 (100.00)	0.3173	NA
	AM group	*	*	0.3173	NA
Convulsion	PV group	0 (0.00)	11107 (100.00)	NA	NA
	AM group	0 (0.00)	11107 (100.00)	NA	NA
Total	PV group	27 (0.24)	11080 (99.76)	0.2562	0.75 (0.45–1.24)
	AM group	36 (0.32)	11071 (99.68)	0.2562	0.75 (0.45–1.24)

* For one or two cases, the number of cases was masked to protect personal information in accordance with the terms of use of the Pediatric Medical Information Collection System (P-MICS) database. AEs, adverse events; PV, pivoxil-conjugated antibody; AM, amoxicillin; NA, not applicable; OR, odds ratio; IC, confidence interval.

## Data Availability

The data presented in this study are available on request from the corresponding author. The data are not publicly available due to protection of personal information.

## Supplementary Materials

The following supporting information can be downloaded at https://www.mdpi.com/article/10.3390/children11020150/s1, Table S1: List of preexisting diseases (specified pediatric chronic diseases and diseases of potentially impacting carnitine deficiency occurrence), Table S2: List of medications potentially impacting carnitine deficiency occurrence, Table S3: All baseline patient characteristics.
