# Supplementary material for: Research on the Clinical Practical Use of Pivoxil-Conjugated Antibodies and the Risk of Carnitine Deficiency Using Real-World Data
Source: Children (Basel). 2024 Jan 24;11(2):150. doi: 10.3390/children11020150 (PMC10887142; doi:10.3390/children11020150)
Supplement: Supplementary file 1 [file children-11-00150-s001.zip › children-2835318-supplementary.pdf]

Table S1: List of preexisting diseases (specific pediatric chronic diseases and diseases of potentially impacting carnitine deficiency occurrence)

Disease name	ICD10 code
Malignant neoplasm	C089, C119, C220, C222, C259, C349, C37, C419, C439, C479, C499, C56, C58, C629, C64, C692, C716, C719, C720, C729, C73, C740, C741, C749, C809, C819, C835, C846, C851, C859, C910, C918, C920, C921, C924, C925, C930, C931, C933, C940, C942, C947, C959, C966, D169, D329, D330, D350, D352, D361, D432, D434, D444, D445, D469, D489, D761, D763
Chronic kidney disease (specified pediatric chronic diseases)	D410, D593, D690, D696, E268, E720, E790, E850, I150, I770, I823, M313, M317, M321, N017, N019, N028, N039, N040, N048, N049, N052, N053, N055, N078, N078, N119, N133, N137, N170, N189, N202, N258, N26, N280, N288, Q273, Q602, Q604, Q605, Q606, Q613, Q614, Q615, Q620, Q622, Q627, Q639, Q649, Q872, Q878
Chronic kidney disease (diseases of potentially impacting carnitine deficiency occurrence)	N030, N039, N110, N118, N119, N181, N182, N184, N185, N189
Chronic respiratory disease	E831, E849, G473, J386, J392, J398, J448, J459, J47, J840, J841, J980, J988, P279, Q330, Q348, Q790, Q878
Chronic heart disease	D487, I495, I050, I070, I071, I209, I219, I251, I253, I270, I279, I280, I288, I311, I319, I340, I348, I350, I351, I370, I371, I420, I422, I424, I425, I428, I441, I442, I454, I456, I458, I471, I472, I489, I490, I493, I514, I719, I770, I971, M303, Q200, Q201, Q202, Q203, Q204, Q205, Q210, , Q212, Q213, Q214, Q221, Q222, Q223, Q224, Q225, Q228, Q230, Q231, Q232, Q233, Q234, Q242, Q243, Q244, Q245, Q248, Q250, Q251, Q253, Q254, Q255, Q256, Q257, Q262, Q263, Q890
Endocrine disorders	C254, D350, D377, D448, E031, E039, E049, E050, E059, E063, E078, E161, E201, E209, E213, E220, E221, E222, E228, E230, E232, E240, E243, E248, E249, E250, E258, E260, E270, E271, E274, E280, E281, E282, E283, E291, E301, E310, E340, E343, E345, E550, E833, E870, E871, E878, E881, E892, I152, N251, Q503, Q551, Q560, Q561, Q562, Q781, Q871, Q878, Q891, Q892, Q969, Q998
Collagen disease	D686, D898, D899, E850, F069, M0800, M082, M0820, M0830, M084, M088, M089, M0890, M300, M301, M313, M314, M317, M320, M321, M329, M330, M331, M332, M339, M349, M350, M351, M352, M9410, P008
Uropathy	E10, E11, E11, E13, E14, E348, E881, P702
Inborn error of metabolism	E701, D528, D818, E701, E702, E708, E710, E711, E713, E720, E721, E722, E723, E724, E725, E728, E729, E740, E741, E742, E744, E748, E749, E750, E751, E752, E754, E755, E760, E761, E762, E770, E771, E780, E783, E784, E786, E788, E789, E791, E798, E799, E802, E830, E832, E880, E888, G241, G318, G601, H498, Q796
Blood dyscrasias	D531, D45, D473, D474, D550, D552, D559, D569, D571, D580, D582, D588, D589, D591, D591, D594, D595, D596, D599, D609, D610, D611, D612, D613, D619, D643, D644, D66, D67, D680, D682, D685, D689, D691, D693, D694, D695, D696, D731, D750, E880, M311
Immune disorders	B24, B372, C845, D70, D71, D721, D729, D763, D800, D802, D803, D805, D806, D807, D809, D810, D813, D815, D816, D817, D818, D820, D821, D823, D824, D828, D839, D841, D848, D849, E703, E803, G113, Q824, Q828, Q938, T860
Neuromuscular disorders	D177, D180, D481, D898, E348, E728, E748, E752, F842, G048, G122, G230, G232, G241, G318, G319, G35, G378, G403, G404, G405, G600, G608, G618, G700, G710, G711, G712, G819, G934, I675, L814, O998, P350, P351, P352, P371, Q019, Q031, Q039, Q042, Q043, Q044, Q046, Q048, Q059, Q281, Q282, Q283, Q750, Q750, Q751, Q851, Q858, Q859, Q870, Q871, Q992
Chronic gastrointestinal disease	D126, D180, D848, E739, E743, E788, E805, E831, I890, K509, K519, K529, K633, K710, K720, K746, K754, K766, K830, K861, K908, K918, Q265, Q266, Q431, Q437, Q438, Q442, Q444, Q445, Q447, Q641, Q858, R11

Disease name	ICD10 code
Syndromes associated with chromosomal or genetic changes	Q783, Q823, Q870, Q871, Q872, Q873, Q874, Q875, Q878, Q898, Q909, Q913, Q917, Q933, Q934, Q935, Q938, Q999
Skin disorders	E703, L401, L511, L512, M8949, Q803, Q804, Q808, Q809, Q819, Q821, Q824, Q850, Q871
Bone system disease	E833, M6119, Q678, Q773, Q774, Q778, Q779, Q780, Q782, Q789, Q872, Q874
Vascular disease	D181, D694, I780, M8950, Q273, Q278, Q820, Q828, Q872
Organic acid metabolism disorder	D818, E711, E711, E713, E723
Fatty acid oxidation disorders	E713
Metabolic disorders associated with Fanconi syndrome	D610, E702, E720
Mitochondrial Disorders	E888
Cirrhosis	B181, B182, I982, I983, K717, K743, K744, K745, K746, K754, K761, K766, P788
Low birth weight infant	P070, P071a, P071b
Malnutrition	E12, E46, D513
Anorexia	F500
Swallowing function disorder	R198
Short bowel syndrome	K918
Coeliac disease	K900
Crohn's disease	D518, K500, K501, K508, K509
Cystic fibrosis	E849
Failure to thrive	R628
Sepsis	A021, A207, A227, A241, A267, A282, A327, A394, A400, A401, A402, A403, A408, A409, A410, A411, A412, A413, A414, A415, A418, A419, A427, A548, B007, B349, B377, D71, I301, I330, J020, J170, J209, J950, L029, R02, R572, U821
AIDS	B210, B220, B24, P002, R75
Renal tubular acidosis	N258
Epidermolysis bullosa	L123, Q810, Q811, Q812, Q818, Q819

Table S2. List of medications potentially impacting carnitine deficiency occurrence

Drug name	ATC code
Quinidine Sulfate Hydrate	C01BA01
Verapamil Hydrochloride	C08DA01
Silver sulfadiazine	D06BA01
Ampicillin Hydrate	J01CA01
Ampicillin Sodium	J01CA01
Bacampicillin Hydrochloride	J01CA06
Piperacillin the product	J01CA12
Ampicillin, combination	J01CA51
Penicillin G	J01CE01
Ampicillin and beta-lactamase inhibitors	J01CR01
Sultamicillin tosylate hydrate	J01CR04
Ampicillin / Cloxacillin	J01CR50
Cephalexin	J01DB01
Cefalotin the product	J01DB03
Cefazolin the product Hydrate	J01DB04
Cefroxadine Hydrate	J01DB11
Cefuroxime Axetil	J01DC02
Cefaclor	J01DC04
Cefotiam Hydrochloride	J01DC07
Cefmetazole the product	J01DC09
Cefminox the product Hydrate	J01DC12
Flomoxef the product	J01DC14
Cefotaxime the product	J01DD01
Ceftazidime Hydrate	J01DD02
Ceftriaxone the product Hydrate	J01DD04
Cefmenoxime Hydrochloride	J01DD05
Latamoxef the product	J01DD06
Ceftizoxime the product	J01DD07
Cefixime Hydrate	J01DD08
Cefpodoxime proxetil	J01DD13
Cefdinir	J01DD15
Cefepime Dihydrochloride Hydrate	J01DE01
Cefozopran Hydrochloride	J01DE03
Aztreonam	J01DF01
Meropenem Hydrate	J01DH02
Doripenem Hydrate	J01DH04
Biapenem	J01DH05
Faropenem the product Hydrate	J01DI03
Ampicillin sodium / sulbactam sodium	J01RA01
Ampicillin / Cloxacillin	J01RA01
Tazobactam / piperacillin	J01RA01
Zidovudine	J05AF01
Zidovudine / Lamivudine	J05AR01
Cyclophosphamide Hydrate	L01AA01
Melphalan	L01AA03
Ifosfamide	L01AA06
Bendamustine Hydrochloride	L01AA09
Bendamustine Hydrochloride hydrate	L01AA09
Busulfan	L01AB01
Thiotepa	L01AC01

Drug name	ATC code
Carmustine	L01AD01
Streptozocin	L01AD04
Nimustine Hydrochloride	L01AD06
Ranimustine	L01AD07
Temozolomide	L01AX03
Dacarbazine	L01AX04
Doxorubicin hydrochloride	L01DB01
Daunorubicin Hydrochloride	L01DB02
Epirubicin Hydrochloride	L01DB03
Aclarubicin Hydrochloride	L01DB04
Idarubicin Hydrochloride	L01DB06
Pirarubicin hydrochloride	L01DB08
Amrubicin Hydrochloride	L01DB10
Cisplatin	L01XA01
Carboplatin	L01XA02
Oxaliplatin	L01XA03
Estramustine Phosphate the product Hydrate	L01XX11
Bupivacaine Hydrochloride Hydrate	N01BB01
Phenobarbital	N03AA02
Phenobarbital the product	N03AA02
Phenytoin	N03AB02
Fosphenytoin the product Hydrate	N03AB05
Phenytoin / phenobarbital	N03AB52
Carbamazepine	N03AF01
Valproic acid	N03AG01

Table S3. All baseline patient characteristics.

Characteristics	Original cohort (n = 47,004)				Matched cohort (n = 22,214)			
	PV group (n = 19,110)	AM group (n = 27,894)	P-value	Std diff	PV group (n = 11,107)	AM group (n = 11,107)	P-value	Std diff
Site category								
Hospital (%)	3,582 (18.74)	20,469 (73.38)	<0.0001	−1.310	3,500 (31.51)	3,713 (33.43)	0.0023	−0.046
Clinic (%)	15,528 (81.26)	7,425 (26.62)	<0.0001	1.310	7,607 (68.49)	7,394 (66.57)	0.0023	0.046
Gender								
Male (%)	10,271 (53.75)	15,896 (56.99)	<0.0001	−0.065	6,485 (58.39)	6,134 (55.23)	<0.0001	0.064
Female (%)	8,839 (46.25)	11,998 (43.01)	<0.0001	0.065	4,622 (41.61)	4,973 (44.77)	<0.0001	−0.064
Age								
Mean (SD)	5.61 (3.95)	4.56 (3.56)	<0.0001	0.279	6.21 (4.11)	5.15 (3.70)	<0.0001	0.279
Minimum	0	0	<0.0001	0.279	0	0	<0.0001	0.279
Maximum	14	14	<0.0001	0.279	14	14	<0.0001	0.279
Newborn (%)	5 (0.03)	41 (0.15)	<0.0001	−0.041	5 (0.05)	3 (0.03)	<0.0001	0.006
Infant (%)	1,032 (5.40)	2,365 (8.48)	<0.0001	−0.121	606 (5.46)	680 (6.12)	<0.0001	−0.026
Toddler (%)	10,782 (56.42)	17,976 (64.44)	<0.0001	−0.165	5,594 (50.36)	6,724 (60.54)	<0.0001	−0.209
Children (%)	7,291 (38.15)	7,512 (26.93)	<0.0001	0.241	4,902 (44.13)	3,700 (33.31)	<0.0001	0.233
Number of clinical site visits								
Mean (SD)	3.19 (7.44)	4.50 (10.45)	<0.0001	−0.144	3.83 (8.76)	4.05 (8.67)	0.0552	−0.025
Minimum	0	0	<0.0001	−0.144	0	0	0.0552	−0.025
Maximum	176	180	<0.0001	−0.144	171	170	0.0552	−0.025
Number of prescribed drugs								
Mean (SD)	4.57 (6.15)	5.07 (8.19)	<0.0001	−0.069	5.33 (6.73)	5.09 (7.56)	0.0134	0.033
Minimum	0	0	<0.0001	−0.069	0	0	0.0134	0.033
Maximum	108	116	<0.0001	−0.069	96	116	0.0134	0.033
Number of disease names								
Mean (SD)	2.52 (3.04)	3.62 (4.71)	<0.0001	−0.279	2.82 (3.30)	2.93 (3.22)	0.0096	−0.029
Minimum	0	0	<0.0001	−0.279	0	0	0.0096	−0.029
Maximum	49	65	<0.0001	−0.279	49	47	0.0096	−0.029
Disease								
Fanconi	0 (0.00)	*	0.2418	−0.012	0 (0.00)	0 (0.00)	NA	0.000
AIDS	0 (0.00)	15 (0.05)	0.0013	−0.033	0 (0.00)	0 (0.00)	NA	0.000
Carnitine palmitoyltransferase	0 (0.00)	0 (0.00)	NA	0.000	0 (0.00)	0 (0.00)	NA	0.000
Crohn's disease	6 (0.03)	13 (0.05)	0.4204	−0.008	4 (0.04)	6 (0.05)	0.5270	−0.009
Propionic acidaemia	0 (0.00)	0 (0.00)	NA	0.000	0 (0.00)	0 (0.00)	NA	0.000
Methylmalonic acidaemia	0 (0.00)	*	0.2418	−0.012	0 (0.00)	0 (0.00)	NA	0.000
Malignant neoplasm	36 (0.19)	200 (0.72)	<0.0001	−0.079	31 (0.28)	43 (0.39)	0.1623	−0.016
Malnutrition	5 (0.03)	52 (0.19)	<0.0001	−0.049	4 (0.04)	7 (0.06)	0.3656	−0.008
Cirrhosis	4 (0.02)	25 (0.09)	0.0032	−0.029	4 (0.04)	5 (0.05)	0.7388	−0.004
Blood dyscrasi	18 (0.09)	101 (0.36)	<0.0001	−0.056	18 (0.16)	19 (0.17)	0.8693	−0.002
Bone system disease	0 (0.00)	18 (0.06)	0.0004	−0.036	0 (0.00)	0 (0.00)	NA	0.000
Anorexia	*	*	0.7879	0.002	*	*	1.0000	0.000
Neuromuscular disorders	41 (0.21)	140 (0.50)	<0.0001	−0.048	35 (0.32)	43 (0.39)	0.3642	−0.012

Renal tubular acidosis	0 (0.00)	4 (0.01)	0.0978	−0.017	0 (0.00)	0 (0.00)	NA	0.000
Swallowing dysfunction	0 (0.00)	0 (0.00)	NA	0.000	0 (0.00)	0 (0.00)	NA	0.000
Inborn error of metabolism	9 (0.05)	71 (0.25)	<0.0001	−0.053	9 (0.08)	9 (0.08)	1.0000	0.000
Syndromes associated with chromosomal or genetic changes	23 (0.12)	171 (0.61)	<0.0001	−0.082	22 (0.20)	22 (0.20)	1.0000	0.000
Short bowel syndrome	0 (0.00)	3 (0.01)	0.1517	−0.015	0 (0.00)	0 (0.00)	NA	0.000
Medium-chain acyl-CoA dehydrogenase deficiency	0 (0.00)	0 (0.00)	NA	0.000	0 (0.00)	0 (0.00)	NA	0.000
Low birth weight infant	4 (0.02)	36 (0.13)	<0.0001	−0.040	3 (0.03)	5 (0.05)	0.4794	−0.007
Diabetes mellitus	44 (0.23)	108 (0.39)	0.0032	−0.028	42 (0.38)	39 (0.35)	0.7384	0.005
Endocrine disorders	103 (0.54)	484 (1.74)	<0.0001	−0.113	99 (0.89)	107 (0.96)	0.5755	−0.007
Cystic fibrosis	0 (0.00)	*	0.4078	−0.008	0 (0.00)	0 (0.00)	NA	0.000
Sepsis	85 (0.44)	1,319 (4.73)	<0.0001	−0.272	82 (0.74)	105 (0.95)	0.0912	−0.013
Failure to thrive	0 (0.00)	1 (0.00)	0.4078	−0.008	0 (0.00)	0 (0.00)	NA	0.000
Skin disorders	*	6 (0.02)	0.3673	−0.009	*	*	1.0000	0.000
Epidermolysis bullosa	*	0 (0.00)	0.2270	0.010	0 (0.00)	0 (0.00)	NA	0.000
Multiple carboxylase deficiency	0 (0.00)	0 (0.00)	NA	0.000	0 (0.00)	0 (0.00)	NA	0.000
Chronic respiratory disease	979 (5.12)	1,561 (5.60)	0.0258	−0.021	625 (5.63)	575 (5.18)	0.1378	0.020
Chronic gastrointestinal disease	33 (0.17)	103 (0.37)	<0.0001	−0.038	25 (0.23)	27 (0.24)	0.7813	−0.003
Chronic heart disease	46 (0.24)	421 (1.51)	<0.0001	−0.137	44 (0.40)	51 (0.46)	0.4717	−0.007
Chronic kidney disease (affect the occurrence of carnitine deficiency)	15 (0.08)	78 (0.28)	<0.0001	−0.048	15 (0.14)	20 (0.18)	0.3977	−0.011
Chronic kidney disease (specified pediatric chronic diseases)	117 (0.61)	493 (1.77)	<0.0001	−0.107	105 (0.95)	127 (1.14)	0.1465	−0.018

Vascular disease	7 (0.04)	11 (0.04)	0.8787	−0.001	5 (0.05)	5 (0.05)	1.0000	0.000
Immune disorders	35 (0.18)	147 (0.53)	<0.0001	−0.058	30 (0.27)	27 (0.24)	0.6907	0.005
Collagen disease	31 (0.16)	167 (0.60)	<0.0001	−0.071	29 (0.26)	31 (0.28)	0.7960	−0.003
Drug								
Aztreonam	0 (0.00)	*	0.2418	−0.012	0 (0.00)	0 (0.00)	NA	0.000
Ampicillin	78 (0.41)	1,988 (7.13)	<0.0001	−0.358	73 (0.66)	77 (0.69)	0.7431	−0.002
Ampicillin, combination	96 (0.50)	1,341 (4.81)	<0.0001	−0.270	96 (0.86)	106 (0.95)	0.4797	−0.006
Idarubicin	*	10 (0.04)	0.0330	−0.021	*	*	1.0000	0.000
Ifosfamide	12 (0.06)	16 (0.06)	0.8125	0.002	10 (0.09)	6 (0.05)	0.3171	0.015
Carbamazepine	26 (0.14)	152 (0.54)	<0.0001	−0.070	24 (0.22)	31 (0.28)	0.3446	−0.011
Carboplatin	10 (0.05)	11 (0.04)	0.5159	0.006	7 (0.06)	6 (0.05)	0.7814	0.004
Quinidine	0 (0.00)	0 (0.00)	NA	0.000	0 (0.00)	0 (0.00)	NA	0.000
Cyclophosphamide	25 (0.13)	86 (0.31)	<0.0001	−0.038	21 (0.19)	25 (0.23)	0.5549	−0.008
Cisplatin	7 (0.04)	14 (0.05)	0.4944	−0.007	6 (0.05)	8 (0.07)	0.5929	−0.009
Sultamicillin	5 (0.03)	10 (0.04)	0.5636	−0.006	4 (0.04)	4 (0.04)	1.0000	0.000
Silver sulfadiazine	0 (0.00)	6 (0.02)	0.0426	−0.021	0 (0.00)	0 (0.00)	NA	0.000
Cefaclor	117 (0.31)	383 (0.69)	0.0003	−0.114	111 (0.50)	120 (0.54)	0.5973	−0.011
Cefazolin	514 (2.69)	1,896 (6.80)	<0.0001	−0.194	495 (4.46)	516 (4.65)	0.4990	−0.009
Cephalexin	13 (0.07)	247 (0.89)	<0.0001	−0.119	13 (0.12)	24 (0.22)	0.0703	−0.014
Cefixime	*	*	0.3590	0.008	*	*	0.5637	0.011
Cefepime	38 (0.20)	138 (0.49)	<0.0001	−0.050	30 (0.27)	28 (0.25)	0.7926	0.003
Cefozopran	*	7 (0.03)	0.2602	−0.011	2 (0.02)	2 (0.02)	1.0000	0.000
Cefotaxime	67 (0.35)	783 (2.81)	<0.0001	−0.198	63 (0.57)	72 (0.65)	0.4372	−0.007
Cefdinir	459 (2.40)	155 (0.56)	<0.0001	0.153	327 (2.94)	130 (1.17)	<0.0001	0.147
Ceftazidime	13 (0.07)	33 (0.12)	0.0868	−0.016	12 (0.11)	17 (0.15)	0.3528	−0.015
Ceftizoxime	3 (0.02)	0 (0.00)	0.0364	0.018	0 (0.00)	0 (0.00)	NA	0.000
Ceftriaxone	75 (0.39)	126 (0.45)	0.3336	−0.009	56 (0.50)	46 (0.41)	0.3210	0.014
Cefpodoxime	15 (0.08)	29 (0.10)	0.3751	−0.008	14 (0.13)	19 (0.17)	0.3837	−0.015
Cefmetazole	161 (0.84)	485 (1.74)	<0.0001	−0.079	154 (1.39)	197 (1.77)	0.0207	−0.034
Cefmenoxime	5 (0.03)	28 (0.10)	0.0028	−0.030	5 (0.05)	10 (0.09)	0.1966	−0.018
Cefroxadine	*	0 (0.00)	0.0875	0.014	0 (0.00)	0 (0.00)	NA	0.000
Daunorubicin	*	29 (0.10)	0.0001	−0.039	*	*	1.0000	0.000
Thiotepa	0 (0.00)	0 (0.00)	NA	0.000	0 (0.00)	0 (0.00)	NA	0.000
Temozolomide	0 (0.00)	*	0.4078	−0.008	0 (0.00)	0 (0.00)	NA	0.000
Doxorubicin	5 (0.03)	27 (0.10)	0.0039	−0.029	5 (0.05)	*	0.2568	0.011
Doripenem	4 (0.02)	*	0.0733	0.016	*	*	0.5637	0.008
Valproic acid	42 (0.22)	265 (0.95)	<0.0001	−0.096	41 (0.37)	48 (0.43)	0.4572	−0.008
Piperacillin	59 (0.31)	67 (0.24)	0.1580	0.013	47 (0.42)	49 (0.44)	0.8379	−0.003
Pirarubicin	11 (0.06)	26 (0.09)	0.1759	−0.013	10 (0.09)	13 (0.12)	0.5314	−0.010
Faropenem	166 (0.87)	24 (0.09)	<0.0001	0.114	55 (0.50)	21 (0.19)	<0.0001	0.044
Phenytoin	*	4 (0.01)	0.3470	−0.009	*	*	0.5637	−0.009
Phenobarbital	40 (0.21)	468 (1.68)	<0.0001	−0.152	39 (0.35)	47 (0.42)	0.3874	−0.007
Busulfan	4 (0.02)	3 (0.01)	0.3745	0.008	4 (0.04)	*	0.4142	0.014
Flomoxef	115 (0.60)	38 (0.14)	<0.0001	0.077	76 (0.68)	33 (0.30)	<0.0001	0.064
Combination with penicillins and other	28 (0.15)	117 (0.42)	<0.0001	−0.051	24 (0.22)	32 (0.29)	0.2844	−0.014

antibacterial agents								
Verapamil	*	0 (0.00)	0.2270	0.010	0 (0.00)	0 (0.00)	NA	0.000
Penicillin G	6 (0.03)	4 (0.01)	0.2130	0.011	*	4 (0.04)	0.4142	−0.012
Fosphenytoin	0 (0.00)	55 (0.20)	<0.0001	−0.063	0 (0.00)	0 (0.00)	NA	0.000
Melphalan	12 (0.06)	5 (0.02)	0.0120	0.022	8 (0.07)	4 (0.04)	0.2481	0.018
Meropenem	33 (0.17)	69 (0.25)	0.0874	−0.016	29 (0.26)	34 (0.31)	0.5281	−0.010

*For one or two cases, the number of cases was masked to protect personal information in accordance with the terms of use of the Pediatric Medical Information Collection System (P-MICS) database. SD, standard deviation; PV, pivoxil-conjugated antibodies; AM, amoxicillin; Std diff, standardized difference.
